# Sonic Hedgehog Signaling Promotes Peri-Lesion Cell Proliferation and Functional Improvement after Cortical Contusion Injury

**DOI:** 10.1089/neur.2020.0016

**Published:** 2021-01-22

**Authors:** Ashley K. Pringle, Elshadaie Solomon, Benjamin J. Coles, Brandon R. Desousa, Anan Shtaya, Shyam Gajavelli, Nedal Dabab, Malik J. Zaben, Diederik O. Bulters, M. Ross Bullock, Aminul I. Ahmed

**Affiliations:** ^1^ Clinical Neurosciences, Faculty of Medicine, University of Southampton, Southampton, United Kingdom.; ^2^ Miami Project to Cure Paralysis, University of Miami, Miami, Florida, USA.; ^3^ Neurosciences Research Centre, St. George's, University of London, London, United Kingdom.; ^4^ Neuroscience and Mental Health Research Institute, University of Cardiff, Cardiff, Wales, United Kingdom.; ^5^ Wessex Neurological Centre, University Hospitals Southampton NHS Trust, Southampton, United Kingdom.; ^6^ Brain Repair and Rehabilitation, Institute of Neurology, London, United Kingdom.

**Keywords:** cerebral cortex, endogenous stem cells, motor recovery, sonic hedgehog, traumatic brain injury

## Abstract

Traumatic brain injury (TBI) is a leading cause of death and disability globally. No drug treatments are available, so interest has turned to endogenous neural stem cells (NSCs) as alternative strategies for treatment. We hypothesized that regulation of cell proliferation through modulation of the sonic hedgehog pathway, a key NSC regulatory pathway, could lead to functional improvement. We assessed sonic hedgehog (Shh) protein levels in the cerebrospinal fluid (CSF) of patients with TBI. Using the cortical contusion injury (CCI) model in rodents, we used pharmacological modulators of Shh signaling to assess cell proliferation within the injured cortex using the marker 5-Ethynyl-2’-deoxyuridine (EdU); 50mg/mL. The phenotype of proliferating cells was determined and quantified. Motor function was assessed using the rotarod test. In patients with TBI there is a reduction of Shh protein in CSF compared with control patients. In rodents, following a severe CCI, quiescent cells become activated. Pharmacologically modulating the Shh signaling pathway leads to changes in the number of newly proliferating injury-induced cells. Upregulation of Shh signaling with Smoothened agonist (SAG) results in an increase of newly proliferating cells expressing glial fibrillary acidic protein (GFAP), whereas the Shh signaling inhibitor cyclopamine leads to a reduction. Some cells expressed doublecortin (DCX) but did not mature into neurons. The SAG-induced increase in proliferation is associated with improved recovery of motor function. Localized restoration of Shh in the injured rodent brain, via increased Shh signaling, has the potential to sustain endogenous cell proliferation and the mitigation of TBI-induced motor deficits albeit without the neuronal differentiation.

## Introduction

Traumatic Brain Injury (TBI) accounts for half of injury-induced deaths^1^ worldwide and is expected to increase over the next decade. With no effective restorative therapies currently available,^2,3^ interest has turned toward neural stem cells (NSCs)/progenitor cells, as they can dynamically sense the injured microenvironment, and can respond with a multitude of restorative factors that alter the injury milieu. Targeting the endogenous NSCs by altering them to modify the injury milieu^4^ may improve the outcome after TBI. Therefore, understanding the NSC response to TBI has the potential to identify new therapeutic strategies for the treatment of TBI.

Endogenous neurogenesis occurs in the hippocampus and the subventricular zone (SVZ) throughout life.^5,6^ Quiescent precursor cells are present in non-permissive regions of the nervous system such as the cortex of rodents^7–10^ and humans.^11^ Within these regions, a TBI induces neurogenesis of the normally quiescent precursor cells.^12–14^ Using an organotypic stretch injury model for TBI, we have shown that after injury equivalent to a severe TBI, a subset of endogenous cells acquired stem cell properties in the injured cortex.^12^ These cells were endogenous to the cortex; they did not migrate from the SVZ.

Injury activates a population of endogenous cells that display NSC properties. However, to harness these cells for repair, the mechanistic pathways that contribute to the process need to be determined. Manipulation of these pathways alters the proliferation of the NSCs, which could lead to improved function. Previously, we demonstrated a transient upregulation of sonic hedgehog (Shh) signaling pathway components in the injured cortex. This was linked with the acquisition of stem cell properties of a subset of cells in the injured cortex.^20^ Shh is a member of the hedgehog family of secreted signaling proteins and has a role in adult stem cell proliferation. In rodents, Shh signaling is active in cells that give rise to neurogenesis in the hippocampus^15,16^ and SVZ.^17–19^ Adult quiescent NSCs proliferate faster in response to Shh^20^ and application of Shh leads to *de novo* neurogenesis in neocortex of adult mice.^21^ In addition to increasing neurogenesis, Shh improves neurite outgrowth of neurons growing on a monolayer of reactive astrocytes.^22^ Thus, Shh acts as a positive regulator of cell proliferation, neurogenesis, and neuronal maturation.

The significance of Shh signaling after injury has been demonstrated. For example, following ischemia there is an increase of Shh in the brain,^23^ and intrathecal administration of Shh in a stroke model has shown a neuroprotective effect.^24^ Similarly, Shh signaling is upregulated in reactive astrocytes following a focal freeze injury to the cerebral cortex.^25^ Moreover, this expression contributes to an increase in the proliferation of a subset of progenitor cells,^25^ suggesting that Shh signaling in the injured cortex directly influences precursor cells. In an *in vitro* model of a cortical astrocytic glial scar, stretch injury leads to impaired neurite outgrowth of neurons placed upon the stretched astrocytic layer, and this was reversed by the addition of Shh.^22^ We have shown in our *in vitro* cortico-hippocampal model that a stretch injury increases expression of components of the Shh signaling pathway.^12^ Therefore, we hypothesized that Shh signaling may contribute to the injury-induced cell proliferation and NSC activation seen following a severe cortical contusion injury (CCI).

Here, we have demonstrated that in patients with TBI, there is a reduction of Shh protein in cerebrospinal fluid (CSF) compared with control patients. In a rodent *in vivo* CCI head injury model, we demonstrated that increasing Shh signaling after injury increases the number of newly proliferating cells in the cortex. This is accompanied by improvement in motor function in rodents. Thus, manipulating the niche environment of the non-permissive injured brain, by increasing Shh signaling, may lead to strategies for endogenous neural repair and subsequent functional recovery.

## Methods

### Shh ELISA in human CSF

CSF samples were collected from patients with severe TBI in which an external ventricular drain was placed for the management of the patient's head injury. Samples were taken from control patients undergoing routine lumbar puncture for neurological investigations, the results of which were normal. Samples were stored at −80°C until analyzed. The Shh concentration was determined by enzyme-linked immunosorbent assay (ELISA; Human Shh N-terminus Quantikine ELISA Kit, DSHH00, Bio-Techne) according to the manufacturer's instructions. All samples were obtained with informed consent and according to guidelines of the National Research Ethics Committee (10/H0502/53).

### Human tissue polymerase chain reaction (PCR)

Normal cortical tissue was resected from five patients during access for epilepsy or tumor surgery. All patients provided informed consent for participation and the human brain tissue samples were anonymized. Ethical approval was obtained from the Research Ethics Committee (12/NW/0 794 and 07/H0504/195). In three cases, hippocampal tissue was also taken from the same patient.

Tissue was transferred in ice-cold artificial CSF (aCSF) and placed directly into TRIzol (Invitrogen). Total RNA was extracted and directly reverse-transcribed to complementary DNA (cDNA) using a Precision qScript RT Kit (Primer Design). The cDNA was amplified using a One-Step PCR Kit (Primer Design) in a real-time thermocycler (Rotor-Gene 6000, Corbett Robotics). Custom-made primers were directed against human Shh, Patched1, and Smoothened (Primer Design; Table 1). Fluorescent data were collected at least once during each cycle of amplification, which allowed for real-time monitoring of the amplification. Data were automatically normalized and a threshold was set at the level where the rate of amplification was the greatest during the exponential phase. Ct-values were collected, and analyzed using the comparative Ct (2[-Delta Delta C(t)], or 2-DDCt) method where the comparative expression level equals to 2-DDCt. The expression of messenger RNA (mRNA) was normalized to a housekeeping gene (β-actin) and expressed as a ratio. Prism Software (Graphpad) was used for data analysis.

**Table 1.** PCR Primer Sequences

Gene	Forward primer	Reverse primer
Shh	GAAGAGGAGGCACCCCAAAA	CCTTAAATCGCTCGGAGTTTCTG
Smo	ACCACCTACCAGCCTCTCTC	CCCACAAAACAAATCCCACTCA
Ptc	GTCGCACAGAACTCCACTCA	GTCAGAGAAGGATTTCAGGATGTC

Primer sequences for RT-qPCR in human neural tissue. Beta-actin primers were obtained from Primer Design (HK-SY-HU-600, ACTB).

PCR, polymerase chain reaction; Ptc, Patched; Shh, sonic hedgehog; Smo, Smoothened.

### The CCI injury and drug treatment

C57Bl/6 mice (20–25 g, 7–8 weeks old) were housed in a 12-h light/dark cycle with food and water *ad libitum*. All animal procedures followed guidelines established by the National Institutes of Health (NIH) Guide for the Care and Use of Laboratory Animals, Animal Research: Reporting of In Vivo Experiments (ARRIVE), and were approved by the University of Miami's Institutional Animal Care and Use Committees (Protocol Number 14-157).

The statistical “power” methods were used to arrive at the appropriate sample size with a power of 0.9 at a 0.05 alpha. A minimum of 10 animals were used in each group for the behavioral analysis and a minimum of 5 animals in each group for the histological analysis. Mice were anesthetized with ketamine (75 mg/kg) and xylazine (14 mg/kg) by intraperitoneal injection and positioned in a stereotaxic frame. Body temperature was monitored and maintained at 37°C. A 5-mm craniotomy was made over the right parieto-temporal cortex (−2.5 mm caudal and 2.0 mm lateral from bregma). The injury was generated using a 3-mm beveled tip attached to a CCI device (Custom Design & Fabrication), at a velocity of 6.0 m/sec, a depth of 0.5 mm, and an impact duration of 150 msec. Sham mice received craniotomy only. After CCI injury the skin was sutured. Starting on the day of injury, mice received the following: 1 — cyclopamine treated animals received intra-peritoneal injection of cyclopamine (10 mg/kg, Sigma Aldrich) diluted in hydroxypropyl beta cyclodextrin (HBC; Sigma Aldrich) once a day for 5 days; 2 - control animals received intra-peritoneal injection of HBC only once a day for 5 days; 3 SAG treated animals received oral administration of SAG (Calbiochem; 0.15 mg in 0.5% methylcellulose/0.2% Tween 80/10 g body weight) once a day for 5 days.

### Behavior

Motor function was tested using the rotarod test. The rotating cylindrical rod was 50-mm wide and had a linear acceleration from 10 rpm to 60 rpm over a period of 10 min (i.e., rate of acceleration was 5 rpm). Mice were pre-trained daily for 1 week prior to the injury. After injury on days 3, 7, and 14, mice were given four trials each day and the time to fall off the rotarod was averaged over the four trials.

### Immunohistochemistry of free-floating sections

Mice were anesthetized using ketamine and xylazine by intraperitoneal injection and transcardially perfused with phosphate-buffered saline (PBS) and 4% paraformaldehyde (pH 7.4). Animals were sacrificed at 7, 14, and 28 days after injury. The brains were stored in 4% paraformaldehyde overnight, 30% sucrose for 48 h, and then transferred into PBS with 0.5% sodium azide. The brains were then sliced coronally around the injury site using a cryostat into 40-μm thick sections. Serial free-floating sections were collected in a 12-well plate. There were approximately four to five free-floating sections that traversed the injury site in each well. The sections were kept in a PBS and 0.5% sodium azide solution at 4°C.

EdU detection was performed using the Click-iT^®^ EdU Alexa Fluor^®^ 594 Imaging Kit following the manufacturer's instructions (C10399, Invitrogen). Sections were subsequently labeled with either GFAP (rabbit, Dako), nestin (mouse, VectorLabs), or DCX (guinea pig, Millipore) pre-diluted in 0.3% Triton X-100 in PBS with 5% donkey serum, with overnight incubation at 4°C. Fluorescent secondary antibodies (Alexa488, Molecular Probes) were incubated for 2 h before further washes. Nuclei were labeled with Hoechst 33342 solution (diluted PBS) before mounting in Mowiol (Harco).

### Imaging and quantification

Cells were imaged at 20 × magnification using a Hamamatsu Orca 1-megapixel CCD camera fitted to a Leica DMIRBE microscope. Images were captured and analyzed using Volocity (Improvision). Four fields were selected at consistent areas of the peri-lesional region corresponding to each corner of the injury site in each of the sections using standard DAPI, fluorescein, and Rhodamine filter sets. To quantify the cell numbers, a 200-μm × 200-μm (40,000 μm^2^) square grid was placed at random within each of the four fields around the peri-lesional area. Cell counts were obtained in each area for Hoechst, EdU, GFAP, and double-labeled cells per 40,000-μm^2^ grid. For three brains in each group at least three sections containing the injury site were analyzed. All images for presentation were taken with a Leica SP5 confocal microscope.

### Statistical analysis

A mixed analysis of variance (ANOVA) model was used to analyze data obtained from the rotarod motor test. Our ANOVA model used treatment groups (SAG, cyclopamine, and control) as a between-subjects factor, and days post-injury (dpi; 0, 3, 7, 14, and 28 dpi) as a within-subjects factor. Mauchly's test was used to evaluate model sphericity. The Bonferroni correction method was used to allow for repeated measures analysis. Independent-samples *t* tests were conducted to study the effect at each time-point. Statistical analyses were completed in IBM SPSS (version 22.0.0.0). Results from the histological studies were analyzed using the Graphpad Prism stats package. Statistical significance was assessed using one-way ANOVA or Kruskal-Wallis test, followed by appropriate post hoc tests.

## Results

### Shh protein levels decrease in the CSF of patients with severe TBI

**FIG. 1.**
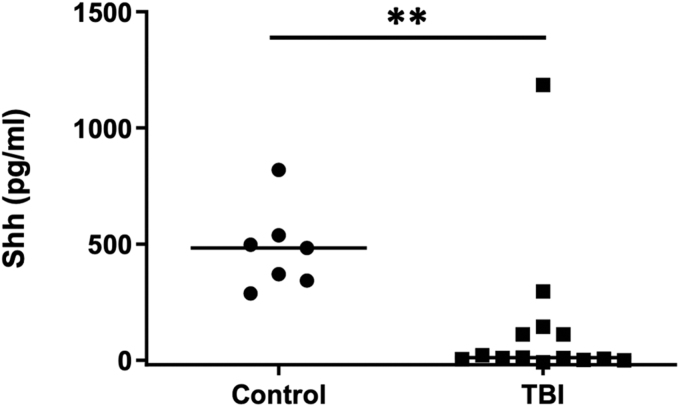
Shh is reduced in CSF following TBI in human patients. CSF was collected from external ventricular drains in patients who had suffered a severe TBI or from a control group undergoing routine lumbar puncture. The concentration of Shh was determined by ELISA and was significantly lower in patients with TBI compared with the control group. Graph shows distribution of the data with the median indicated by the horizontal bars (control, *n* = 7; TBI, *n* = 14; **p < 0.01). CSF, cerebrospinal fluid; ELISA, enzyme-linked immunosorbent assay; Shh, sonic hedgehog; TBI, traumatic brain injury.

There are limited data on the level of Shh in the human central nervous system. To determine if Shh levels were altered by TBI, we compared the concentration of Shh protein in the CSF of patients requiring an external ventricular drain (EVD) following a severe TBI (Glasgow Coma Scale score <8, with raised intracranial pressure necessitating an EVD to relieve that pressure) with CSF of non-TBI patients. CSF from patients with TBI was obtained at a median of 1.5 days from injury (median, 1.5 days; range, 0–3 days). In patients with TBI, the level of Shh in CSF was significantly lower compared with uninjured control patients (10.5 pg/mL vs. 483.1 pg/mL; *p* < 0.01, Mann-Whitney U test; Fig. 1).

Next, we demonstrated that human cortical cells contain Shh signaling components. Patients undergoing either epilepsy surgery or tumor surgery had resected “normal” access tissue frozen and analyzed. Human cDNA from cortical and hippocampal tissue demonstrated expression of Shh and its receptors Smoothened and Patched1. Expression of all three genes was demonstrated in both brain regions with no significant difference in levels between cortex or hippocampus (Table 2).

**Table 2.** Human PCR

	Cortex	Hippocampus
Shh	10.26 ± 1.46	9.33 ± 1.29
Smo	9.90 ± 1.44	9.20 ± 1.42
Ptc	9.63 ± 0.68	9.30 ± 0.44

Relative expression of Shh, Smo, and Ptc in cortex (*n* = 5) and hippocampus (*n* = 3) from adult human brain. Expression of all three genes occurs in both brain regions, with no significant difference observed.

PCR, polymerase chain reaction; Ptc, Patched; Shh, sonic hedgehog; Smo, Smoothened.

### Increasing Shh signaling results in increased cell proliferation

**FIG. 2.**
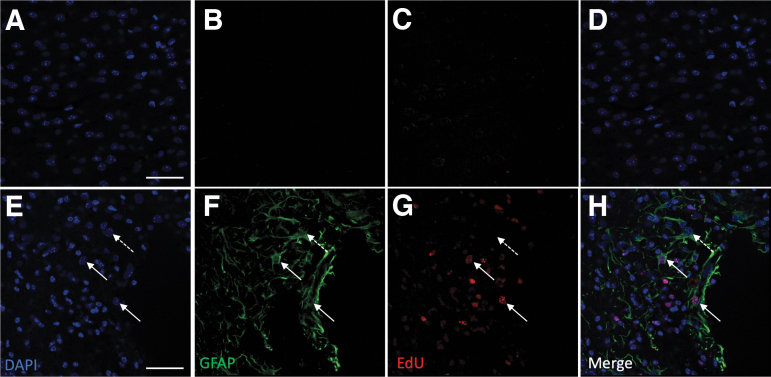
Example images demonstrating injury-induced proliferation in the cortex 7 dpi after CCI. **(A–D)** In the uninjured (contralateral) cortex, DAPI+ cell nuclei are present (A), but there were negligible GFAP+ (B) or EdU+ (C) cells. (D) is a composite of A–C. However, in the area surrounding the injury (ipsilateral cortex), GFAP+ cells and processes were clearly detectable. Proliferating cells, as indicated by EdU staining were also clearly detectable **(G)**. **(H)** A composite of **E–G** and demonstrates that GFAP and EdU staining co-localize in some cells (solid arrows), whereas other cells express GFAP only (dotted arrow). Scale bar = 50 μm. CCI, cortical contusion injury; dpi, days post-injury; EdU, 5-Ethynyl-2’-deoxyuridine; GFAP, glial fibrillary acidic protein.

**FIG. 3.**
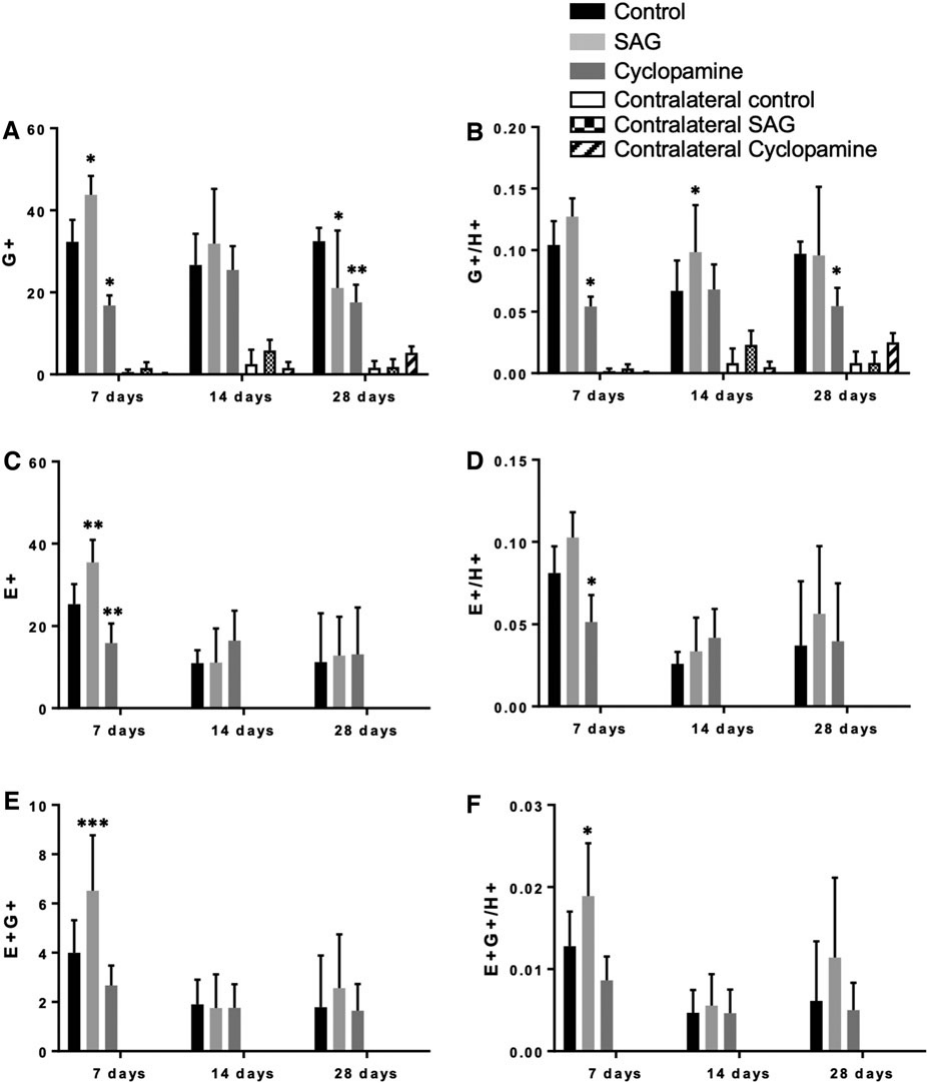
Shh signaling post-injury enhances NSC proliferation. **(A)** GFAP+ (G+) cells were observed in the cortex ipsilateral to the injury (control, SAG, and cyclopamine), but not in the contralateral cortex. In control brains, the number of G+ cells remained unchanged 7–28 days post-injury. At 7 days post-injury there was a significant increase in G+ cells in SAG-treated mice. The opposite effect occurred with cyclopamine treatment. The effect of SAG was reversed by 28 days with a significant decrease in G+ cells being observed. **(B)** A similar effect of cyclopamine was observed when the proportion of GFAP+ cells (G+/H+) was considered, with a reduction in G+/H+ cells occurring at 7 and 28 dpi. In SAG-treated mice, an increase was observed at 14 days post-injury, with no significant effect observed at other time-points. **(C)** The number of proliferating cells was detected by positive EdU labeling (E+). E+ cells were only observed on the side ipsilateral to the injury. SAG induced an increase in E+ cells 7 days post-injury, an effect that was transient and was lost by 14 days post-injury. Conversely, cyclopamine resulted in a decrease in proliferation at 7 days but not later. **(D)** The proportion of E+ cells (E+/H+) was decreased at 7 days by cyclopamine, with no effect at later time-points. There was no significant effect of SAG at any time-point. **(E)** Proliferating NSCs will be both GFAP+ and EdU+ (E+G+). Small numbers of E+G+ cells were detected in the ipsilateral cortex. SAG treatment significantly increased the number of E+G+ cells at 7 dpi, an effect that was lost by later time-points. No effect of cyclopamine on E+G+ cells was seen. **(F)** SAG treatment also increased the proportion of E+G+ cells (E+G+/H+) at 7 days post-injury but not at later time-points. Cyclopamine had no effect on the number of E+G+ cells. (*n* = 4–11; *p < 0.05, **p < 0.01, ***p < 0.001 vs. control injury). dpi, days post-injury; EdU, 5-Ethynyl-2’-deoxyuridine; GFAP, glial fibrillary acidic protein; NSC, neural stem cell; SAG, Smoothened agonist; Shh, sonic hedgehog.

We previously demonstrated that in mice, the mRNA of Shh signaling components is increased after injury.^12^ We therefore sought to determine if the increase in cell proliferation post-injury could be modulated by Shh signaling. Proliferating GFAP-positive (GFAP+) cells may represent either reactive astrocytes or NSCs. CCI induces ipsilateral cells to acquire NSC-like properties *in vivo*, including proliferation to form neurospheres and differentiation to astrocytes, oligodendrocytes, and neurons (Supplementary Appendix S1; Supplementary Figs. S1 and S2). Following a CCI, animals were treated either with the Shh antagonist cyclopamine or the Shh agonist SAG. The phenotype of injury-induced proliferating (EdU+) cells was investigated. Significant numbers of GFAP+ cells were observed up to 28 dpi (Figs. 2 and 3A). At 7 dpi, the total number of GFAP+ cells were significantly increased in SAG-treated animals, and reduced in cyclopamine-treated animals (Fig. 3B, *p* < 0.05), but this effect was lost by 14 dpi. Although changes in the total number of GFAP+ cells give an indication of the effect of SAG and cyclopamine, the total number of cells (Hoescht+) is also affected in each condition. A more accurate reflection is to consider the proportion of the total number of cells that are GFAP+ (GFAP+/Hoescht+). This increased with SAG and decreased with cyclopamine (*p* < 0.05; Fig. 3B).

To determine the number of new injury-induced cells, EdU+ cells were quantified. After 7 dpi, SAG significantly increased proliferation when compared with the control injury group, whereas cyclopamine decreased proliferation (Fig. 3C, *p* < 0.01). Both of these effects were transient, with no effect of either compound observed at 14 dpi or 28 dpi (Fig. 3C). The proportion of the total cells that were proliferating (the mitotic index, Edu+/Hoescht+) was significantly reduced with cyclopamine treatment at 7 dpi (*p* < 0.05), whereas SAG had no effect (Fig. 3D).

To determine the phenotype of the EdU+ cells, we investigated the cells that co-expressed GFAP and Edu (EdU+GFAP+) because some of these cells may represent the NSC population within the cortex. We observed a significant increase in the total number of EdU+GFAP+ cells and the proportion of the total number of GFAP cells that were newly proliferating (EdU+GFAP+/Hoescht+) at 7 dpi in SAG-treated mice (*p* < 0.001). This effect was transient, being lost at 14 dpi and 28 dpi. No significant effect of cyclopamine was observed, although there was a downward trend at 7 dpi (Fig. 3E).

Although early-stage proliferating neural stem/progenitor cells would be expected to be double labeled with EdU and GFAP, this is not a definitive marker. As such, we also investigated the number of newly proliferating cells that expressed the intermediate filament protein nestin. However, at both 7 dpi and 14 dpi, the number of nestin+ cells was low in CCI-injured animals (1.4 ± 0.6 cells per field at 7 dpi; 1.2 ± 0.84 cells/field at 14 dpi), with even fewer cells visible in the SAG or cyclopamine groups.

### Effect of Shh signaling on doublecortin (DCX) expression

**FIG. 4.**
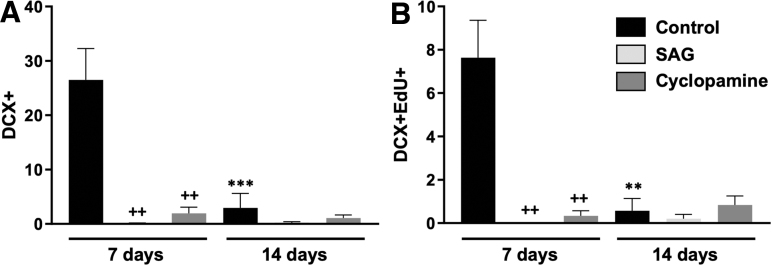
Modulation of DCX expression by CCI. **(A)** The number of DCX+ cells in the peri-injury area was determined by immunohistochemistry 7 days and 14 days after CCI. Following injury alone (control), DCX+ cells were observed in the cortex around the injury 7 days later. By 14 days after injury, the number of DCX+ cells had significantly declined. In mice treated with either SAG or cyclopamine, the number of DCX-positive cells was significantly lower than in control injury mice at 7 days post-injury. **(B)** DCX+ cells derived from proliferating cells were double-labeled with EdU (DCX+EdU+). A small number of DCX+EdU+ cells were found in the cortex around the lesion 7 dpi. As with the DCX+ cells, the number of DCX+EdU+ significantly declined by 14 dpi. Similarly, the number of DCX+EdU+ cells at 7 dpi was significantly lower in mice treated with either SAG or cyclopamine. (*n* = 3, ++*p* < 0.01 vs. control; **p < 0.01 vs. 7 day control; ***p < 0.001 vs. control). CCI, cortical contusion injury; dpi, days post-injury; DCX, doublecortin; . EdU, 5-Ethynyl-2’-deoxyuridine; SAG, Smoothened agonist.

**FIG. 5.**
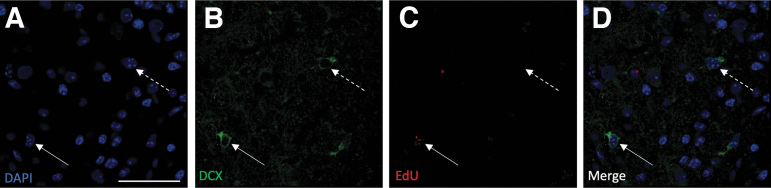
Images showing both DCX+ and DCX+EdU+ cells in the cortex 7 dpi. **(A)** DAPI-staining of cell nuclei (blue); **(B)** immunohistochemistry for DCX (green); **(C)** EdU staining (red); **(D)** composite image indicating cell labeled with DCX only (dashed arrow) or double-labeled with DCX and EdU (solid arrow). Scale bar = 50 μm. dpi, days post-injury; DCX, doublecortin; EdU, 5-Ethynyl-2’-deoxyuridine.

NPCs that move from transiently amplifying progenitor cells toward a more mature neuronal phenotype will downregulate nestin expression, as they gradually express the early neuronal protein DCX.^26^ We therefore determined the number of DCX+ cells at 7 and 14 days post-injury. At 7 dpi, DCX+ cells were observed in the peri-lesional area in the control injury group (26.5 ± 5.8 cells/area), but not in the SAG (0.1 ± 0.1 cells, *p* < 0.01 vs. control) or cyclopamine (1.1 ± 0.6; *p* < 0.01 vs. control) groups. By 14 days, there was a significant decrease in the number of DCX+ cells in the control injury group (2.9 ± 2.7 cells, *p* < 0.001 vs. 7 dpi control) with no expression in either the SAG or cyclopamine groups (Fig. 4A and Fig. 5).When considering the number of DCX+ cells maturing during the peri-lesional period, we determined the number of cells that were double-labeled for both DCX and EdU. Double-labeled cells were observed in the control injury group at 7 dpi (7.6 ± 1.7), but not in the SAG (0 ± 0, *p* < 0.01 vs. control) or cyclopamine (0.3 ± 0.2, *p* < 0.01) treated animals. By 14 dpi, the number of double-labeled cells in the control injury group was reduced (0.6 ± 0.6, *p* < 0.01 vs. 7 dpi control; Fig. 4B).

### Activation of Shh pathway improves functional recovery

**FIG. 6.**
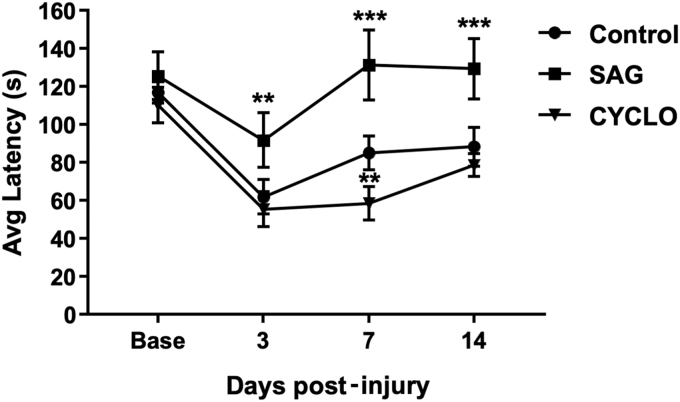
Activation of Shh signaling with SAG significantly improves recovery of motor function after CCI injury. Motor function was determined by the average time mice could remain on a rotarod apparatus. Baseline performance was determined prior to injury after mice had been acclimatized to the rotarod. In the control injury group (circle), average latency was significantly reduced 3 days post-injury and partially recovered toward baseline up to 14 days post-injury. A similar pattern was observed in mice treated with cyclopamine (triangle), although the recovery was slower, remaining significantly behind the control group at 7 dpi. In contrast, mice treated with SAG (square) showed an enhanced recovery that was significantly better than control at all three time-points post-injury and which returned to pre-injury baseline levels by day 7. (*n* = 9–10, **p < 0.01 vs. control; ***p < 0.001 vs. control). CCI, cortical contusion injury; CYCLO, cyclopamine; dpi, days post-injury; SAG, Smoothened agonist; Shh, sonic hedgehog.

We next sought to determine if the transient maintenance of GFAP cells with Shh agonists improved motor function. Motor function was assessed using a rotarod system on which mice had been trained daily for 7 days prior to injury. The mean latency scores after training were equivalent between groups (115 ± 6 sec, control; 125 ± 12 sec, SAG; 110 ± 9 sec, cyclopamine). In control CCI, motor performance was significantly impaired (61 ± 9 sec) 3 days after CCI compared with baseline, with a partial recovery by 14 dpi (88 ± 1 sec). A similar pattern was observed in mice treated with cyclopamine, although there was a trend toward a reduction in recovery with cyclopamine. In contrast, SAG treatment resulted in significantly improved recovery at 3 dpi (91 ± 18 sec) followed by recovery to baseline levels after 7 and 14 dpi (Fig. 6). This suggests that modulation of Shh signaling alters motor recovery post-injury.

## Discussion

Following a CCI, we demonstrated an overall increase in GFAP-expressing cells ipsilateral to the injury. In a previous *in vitro* study, we demonstrated that a proportion of these cells are stem/progenitor cells.^12^ The Shh receptor, Patched1, was upregulated following injury, suggesting that Shh signaling may be important in regulating the proliferation of injury-induced GFAP cells.^12^ In this study, using pharmacological modulators of Shh we demonstrated that activating Shh signaling transiently increases the number of newly proliferating GFAP cells on the injured side, while inhibiting Shh reduces cell proliferation. This suggests that modulating Shh results in changes in the number of injury-induced GFAP cells, and we know from our previous work that a proportion of these injury-induced proliferating cells are NSCs. The GFAP+ cells could be referred to as “reactive astrocytes” and NSCs can be derived from mature astrocytes.^8^ To clarify if these injury-induced GFAP+ cells are different to reactive astrocytes, future studies with cell-type specific reporters, such as nestin or DCX reporter mice, could be used. This would objectively assess the fate of proliferating cells induced by TBI, complementing our histological work.

In line with our findings, several injury models demonstrate an increase in progenitor cells following the injury, and this has been shown to be augmented by the Shh agonist SAG. For instance, in a stab injury model, SAG results in an increase in the number of newly proliferating GFAP+ cells.^27^ Similarly, following an ischemic stroke, progenitors from the SVZ migrating into the ischemic tissue are increased following SAG treatment.^28^ Therefore, in models of injury that lead to cells acquiring stem-like properties at the injury site, activation of Shh signaling by SAG acts to increase the stem/progenitor response. Similarly, migrating GFAP-expressing NSCs from the SVZ are regulated by Shh, and manipulation of the Shh pathway alters the fate of these cells.^17^ This implies that there is a common mechanism of NSC activation by Shh for cells endogenous to the cortex and those that may migrate from the SVZ.

Post-injury, NSCs can arise *de novo* from endogenous precursors or can migrate into the lesion. For example, after an aspiration lesion, DCS+ cells migrate into the injury site from the SVZ.^29^ NSCs that migrate from the SVZ influence the injury microenvironment, suggesting that these migrating NSCs may stabilize the injury milieu after injury.^13^ Their role in limiting the lesion expansion is well documented because stem cell ablation induces lesion expansion.^8,30,31^

Endogenous cells can also contribute to the injury response and this includes GFAP-expressing cells. GFAP is well characterized as a marker of radial glia such as quiescent stem cells, in addition to being a marker of reactive astrocytes.^32,33^ Following a CCI, GFAP+ cells start proliferating^34,35^ and some of these cells also express the stem cell progenitor markers nestin and sox2 in addition to the immature neuronal marker DCX.^34,35^ This increase in GFAP cells may represent both reactive astrocytes and an NSC population because some of these cells are nestin+ and DCX+. After a cortical stab injury that induces a reactive gliosis, fate mapping showed that GFAP cells start proliferating and these cells acquire stem cell-like properties when cultured *in vitro.*^8^ More recently, the same group showed that the stab incision results in endogenous NSC activation, due in part to Shh, and this acts directly on cortical cells leading to increased proliferation.^27^ We have demonstrated that in a rodent *ex vivo* model, endogenous cells are activated after a TBI, display stem cell-like features when cultured *in vitro*,^12^ and their proliferation is affected by modulation of Shh signaling.

We sought to determine if these proliferating cells expressed other markers of stem cells or progenitor cells. Although there were too few cells that were nestin+ to correlate any findings to Shh modulation, it was nevertheless interesting to see nestin expression within the injured cortex in control and treated animals because nestin is a well-established NSC marker. At 7 dpi, DCX, an early neuronal marker, was increased compared with the uninjured contralateral side. A proportion of these cells were newly proliferating because they were labelled with EdU. Although inhibiting Shh reduced the number of DCX-labeled cells significantly at 7 dpi, increasing Shh signaling paradoxically had a similar effect. This may be explained by the SAG accelerating the maturation of the progenitor cells, pushing them out of a state of immaturity to a mature phenotype as indicated by loss of DCX expression,^36^ or perhaps even pushing these cells away from a neuronal phenotype.^37^

Alternatively, altering the Shh pathway could result in increased cell death leading to reduced DCX expression. Although in mammals most DCX+ cells in normal adult neurogenesis undergo cell death, DCX ablation of the remaining cells impedes cognitive recovery.^38^ Further work should be directed at determining the fate of these cells in the longer term beyond 7 dpi, for instance by fate mapping using genetically labeled cells, because only low numbers of labeled cells were observed using our current methodology. Others have demonstrated that GFAP+/sox2+ radial glia cell numbers peak at 3 days following a CCI.^35^ Although the authors suggest these cells migrate from the SVZ, they may also arise from endogenous precursors. Interestingly, retroviral delivery of transcription factors leads to the conversion of reactive glia into stem/progenitor cells and subsequently into neurons following TBI,^39^ suggesting that these cells are amenable to reprogramming. Our studies do not support the notion that these cells become neurons, as the time course is too short to allow this.

Importantly, the effects of modulating Shh signaling on cell proliferation correlated with improved motor recovery. The post-injury rotarod test performance was significantly improved by SAG treatment. This effect was significant compared with CCI-control mice at 3 dpi, and the recovery was maintained until 14 dpi. In contrast, the performance of mice treated with the Shh inhibitor cyclopamine was indistinguishable from controls after 14 dpi. Therefore, we can infer that increasing the number of proliferating GFAP+ cells, through activation of Shh signaling, correlates with improved motor function. Alternative mechanisms include cell death resulting in lesion size expansion or direct modulation of the inflammatory environment by Shh signaling.

The early timing of the motor recovery within days is unlikely a consequence of neuronal integration-based recovery. In the early period, the effect is likely to be a neuroprotective effect of Shh, such as that seen with other pharmacological agents.^40,41^ One way to investigate this would be to use a transgenic approach to genetically ablate the progenitors following SAG treatment to determine if they contribute to the functional improvement observed. Endogenous stem/progenitor cells contribute to functional recovery after several injury models including TBI. Ablating stem/progenitor cells migrating from the SVZ reduces the recovery of motor function following a CCI.^13^ In a middle cerebral artery occlusion stroke model, genetic deletion of Shh expression in nestin-expressing cells led to worsening motor ability.^42^ Further, SAG treatment improved both locomotor function and cognitive function following stroke.^28^ In a nigrostriatal model for neurodegeneration, transplanted NSCs with silenced Shh signaling impaired the neuroprotective effects of the NSCs.^43^ There is therefore strong evidence linking activation of Shh signaling and stem cell activation with improved recovery in several models of neural injury and neurodegeneration.

If we are to translate the observations from our rodent studies into patients, whereby increasing Shh with pharmacological inhibitors could affect endogenous precursors, we need to determine whether Shh is altered following TBI and also if the human brain contains the necessary machinery to respond to modulation of Shh signaling. Importantly, in patients with TBI, we have shown that there is a significant decrease in Shh in the CSF, although why this occurs is not known. Our finding suggests an opportunity to increase Shh in CSF through small-cell activators of Shh signaling. There are known steroid agonists of Shh signaling, currently in clinical use, which could be tested.^44^ Although Shh levels within the parenchyma of patients with TBI is not known, there is a transient decrease in Shh protein levels in the rat brain following a CCI.^45^ In human cortex, the components of the signaling pathway are equivalent in mRNA expression compared with the hippocampus, the latter we know in humans is rich in NSCs.^46^ Interestingly, it has been observed that there is an increase in DCX-expressing cells in brains from TBI patients.^47^ It would therefore be useful to investigate the endogenous progenitor/stem response in patients with TBI, including receptor levels, coupled with *in vitro* human progenitor work to determine if human cells respond to Shh small-molecule activators. This would begin to answer the key clinical question of whether this treatment could improve recovery in patients.

## Conclusion

We demonstrated that increasing Shh signaling after injury increases the number of newly proliferating cells, and this is accompanied by improvement in motor function in rodents. Therefore, we can infer that increasing the number of proliferating GFAP+ cells (a proportion of which are progenitor cells) through activation of Shh signaling offers an avenue to manipulate the niche environment of the non-permissive injured brain. This may lead to strategies for endogenous neural repair and subsequent functional recovery.

## Supplementary Material

Supplemental data

Supplemental data

Supplemental data

## Abbreviations Used

2-DDCt: comparative Ct
aCSF: artificial cerebrospinal fluid
ANOVA: analysis of variance
CCI: cortical contusion injury
cDNA: complementary DNA
CSF: cerebrospinal fluid
DCX: doublecortin
dpi: days post-injury
EdU: 5-Ethynyl-2’-deoxyuridine
ELISA: enzyme-linked immunosorbent assay
EVD: external ventricular drain
GFAP: glial fibrillary acidic protein
HBC: hydroxypropyl beta cyclodextrin
mRNA: messenger RNA
NSC: neural stem cell
PBS: phosphate-buffered saline
PCR: polymerase chain reaction
SAG: Smoothened agonist
Shh: sonic hedgehog
SVZ: subventricular zone
TBI: traumatic brain injury
