# Supplementary material for: Sonic Hedgehog Signaling Promotes Peri-Lesion Cell Proliferation and Functional Improvement after Cortical Contusion Injury
Source: Neurotrauma Rep. 2021 Jan 22;2(1):27–38. doi: 10.1089/neur.2020.0016 (PMC7962778; doi:10.1089/neur.2020.0016)
Supplement: Supplemental data [file Supp_AppendixS1.docx]

**Supplementary Data**

Supplementary Methods

*Neurosphere culture methods*

Mice were anaesthetised using ketamine and xylazine by IP injection. The mouse whole brains were quickly dissected and the mid/hindbrain was discarded. The hemisphere was transferred to a McIlwain chopper and 400µm slices were cut and transferred to ice cold Gey’s balanced salt solution (with 0.45% glucose). In the Gey’s solution, a sharpened tungsten wire was used to dissect out the outer cortex (ensuring the inner subcortical and subventricular zones were not included, Supplementary Figure 1). The tissue was washed once in pre-warmed Neurobasal-A media and incubated in sterile papain solution (2mg/mL in Neurobasal-A with 2% B27) with gentle agitation for 40 min at 37°C. The tissue was washed in neurosphere media (Neurobasal-A, 2% B27, 2mM glutamine, 1% antibiotic, 20ng/mL epidermal growth factor [EGF], 20ng/mL fibroblast growth factor [FGF]-2, heparin 2lg/mL) and gently triturated with a fire-polished glass pipette into suspension. The solution was passed through a 40um cell strainer (Beckton Dickinson) and pipetted onto an OptiPrepTM gradient (10% above a 20% solution) and centrifuged at 1900 rpm for 15 min. The middle cellular layer was removed, resuspended in 2mL media, and centrifuged at 1100 rpm for 2 min. The supernatant was discarded and the pellet resuspended in culture media, assessed for viable cells using the Trypan Blue assay (Sigma) and plated at 50,000 cells/mL in neurosphere media in an incubator at 37°C, 5% CO2. Half of the media was changed twice weekly. At 10 days in vitro (DIV), the number of neurospheres per well (>100um diameter) was determined.

*Generation of secondary clonal spheres*

Individual primary spheres were taken at 10 DIV and mechanically dissociated into a single cell suspension by triturating 20–30 times. The suspension was diluted into a total of 300uL neurosphere media, and after ensuring single cell dissociation, secondary spheres were generated at clonal density after 10–14 DIV. Half the media was changed twice weekly. Individual spheres were taken and plated on glass cover-slips, previously coated with poly-l-lysine (20 ng/mL, Sigma) and then laminin (20ng/mL; Sigma), in differentiation media (Neurobasal-A, 2% B27, 2 mM glutamine, 1% antibiotic, 2% fetal bovine serum). Media was changed twice weekly for 10 DIV before spheres were fixed with 4% paraformaldehyde (PFA) for 15–30 min. After washes and blocking in PBST with blocking serum (PBS, 0.1% triton [BDH], 5% donkey serum [Sigma]), spheres were stained Beta III tubulin (Covance, rabbit), GFAP (Dako, rabbit), and Olig 2 (Millipore) overnight at 4°C. The spheres were incubated with fluorescent secondary antibodies (Alexa, molecular probes) followed by washes. DAPI (Sigma) was applied before mounting in Mowiol (Harco).

Supplementary Results

*CCI induces cells to acquire stem like properties in vivo*

The adult cortex is traditionally regarded as non-neurogenic. Neurogenesis is restricted to the hippocampus and SVZ in the adult rodent. We previously demonstrated that in an *ex vivo* slice culture model for TBI, the injured cortex contains multipotent cells with capacity to proliferate evident upon culturing *in vitro*^12^. We sought to investigate if this occurs *in vivo*. Without injury, there are no proliferating cells in *in vitro* cultures. However, after a CCI, dissociated injured cortex gives rise to neurospheres with a peak at 3 days (p<0.001, t-test). This elevated proliferation persists up to 7dpi (p<0.01), although at a smaller magnitude and is lost by 14 days post injury (p=NS; Supplementary Figure 2).

To demonstrate that the post-injury proliferating cells were stem/progenitor cells, we generated secondary clonal spheres. Primary spheres were dissociated after 10 days and plated at a clonal density. Subsequent secondary spheres were then allowed to differentiate for 10-14 days. Immunohistochemistry confirmed the presence of neurons (β-tubulin), astrocytes (GFAP) and oligodendrocytes (Olig-2) suggested neurospheres were cortical stem/progenitor cells (Supplementary Figure 2B and 2C). The data indicates that post-CCI, cells acquire stem/progenitor properties in the injured cortex, which appear to be transient.

Injured cortical tissue contains cells which acquire the ability to behave as stem/progenitor cells^8, 12^. These cells have two key features, firstly the ability to proliferate *in vitro*, in the presence of growth factors, to form neurospheres, and secondly the ability of these cells to demonstrate multipotency when cultured at a clonal density. Our results suggest that this stem/progenitor potential is transient and has a maximal effect at 3 days and corroborate our previous *ex vivo* slice culture data which also demonstrated that these cells originated from the cortex and acquired stem/progenitor properties, but only following an injury^12^. In the current study, we have shown that a proportion of the cells in the cortex express GFAP. This does raise the possibility that these GFAP expressing cells are similar in characteristics to the radial glia like stem/progenitor cells found in the cortex of the rodent embryonic brain^43^.

*There is no contralateral cell proliferation after a Unilateral CCI*

Amongst rodent models, the CCI is traditionally regarding as a focal injury, while the fluid percussion model is regarded as more diffuse in nature. However, this is dependent on the severity of the injury and parameters of the device which include depth, latency and dwell time of the CCI device^44^. We sought to confirm that any injury-induced proliferation specifically in the cortex was confined to the injured side, so as to allow the contralateral uninjured side to serve as an internal control for any proliferation effects demonstrated. In order to investigate injury-induced proliferation an immunohistochemical approach was used. EdU was used as a marker of proliferating cells and animals received EdU for 5 days after CCI to label all injury-induced proliferating cells. Proliferating cell phenotyping was performed with double labelling for cell markers and Hoechst was used as a counterstain.

In the hemisphere contralateral to the injury, Hoescht+ and Hoescht+EdU+ staining revealed neurons organised into distinct cortical layers consistent with the normal histological appearance of the cortex. On the injured side, CCI resulted in a consistent lesion penetrating into the cortex to just above the lateral ventricles (Supplementary Figure 1). Prior to determining the effects on newly proliferating cells on the ipsilateral injured side following a CCI injury, we sought to confirm that the injury remained focal with effects on proliferation localized around the injury site. EdU is an established marker of cell proliferation and was administered for 5 days after a CCI. On the contralateral side, no EdU cells seen were seen in any experimental conditions (Figure 2A and 3C). While there were a small number of GFAP expressing cells as would be expected within normal uninjured cortex, no markers of progenitor cells (nestin) or immature neurons (DCX) were seen on the contralateral side. This allowed us to use the contralateral side as an internal uninjured control.
