# Supplementary material for: Sonic Hedgehog Signaling Promotes Peri-Lesion Cell Proliferation and Functional Improvement after Cortical Contusion Injury
Source: Neurotrauma Rep. 2021 Jan 22;2(1):27–38. doi: 10.1089/neur.2020.0016 (PMC7962778; doi:10.1089/neur.2020.0016)
Supplement: Supplemental data [file Supp_FigS1.pdf]

Supplementary Data

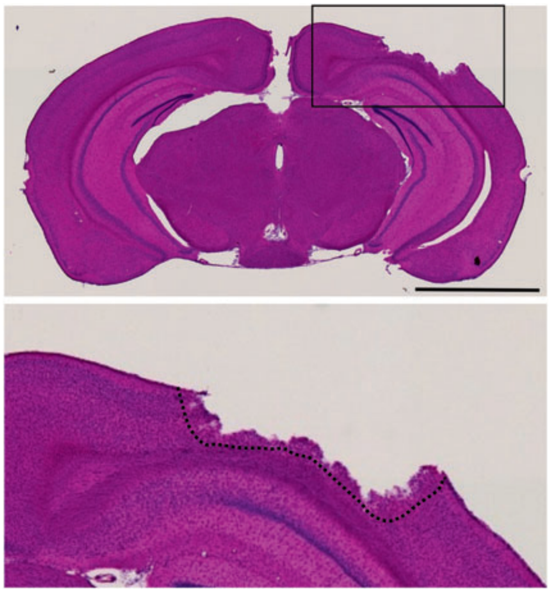

SUPPLEMENTARY FIG S1. **(A)** Coronal section demonstrating the injury site 7 days after CCI (H&E stain). Scale bar = 2 mm. **(B)** Magnified view of the CCI injury area. The dotted line indicates the peri-injury area from which cells were harvested for the neurosphere experiments. CCI, cortical contusion injury.
