# Supplementary material for: Sonic Hedgehog Signaling Promotes Peri-Lesion Cell Proliferation and Functional Improvement after Cortical Contusion Injury
Source: Neurotrauma Rep. 2021 Jan 22;2(1):27–38. doi: 10.1089/neur.2020.0016 (PMC7962778; doi:10.1089/neur.2020.0016)
Supplement: Supplemental data [file Supp_FigS2.pdf]

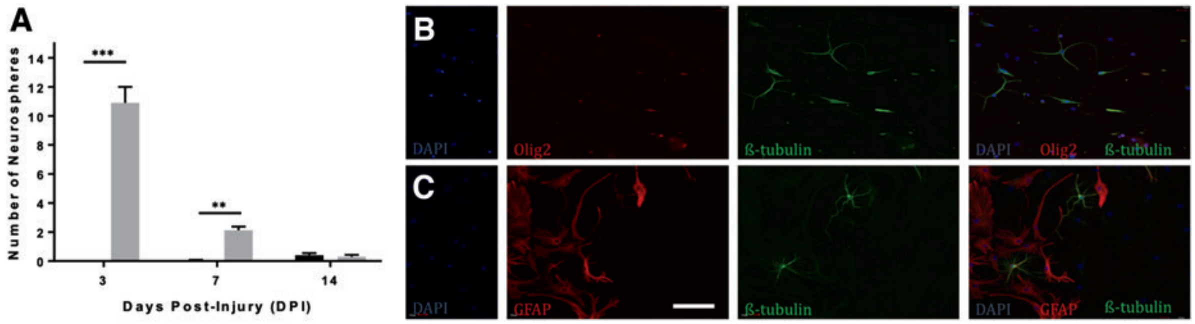

SUPPLEMENTARY FIG. S2. Development of stem cell-like properties in cortical cells following CCI. **(A)** Cells were isolated from the area immediately surrounding the injury site 3, 7, and 14 days post-injury and maintained in culture for 10 days. The number of neurospheres present in each group at 10 DIV was determined. No neurospheres were observed in cultures from uninjured mice at 3 and 7 days with only a very small number being observed at 14 days (black bars). In contrast, in cultures derived from cortex 3 or 7 days after injury, a significant increase in the number of neurospheres was observed, declining to basal levels at 14 days post-injury (gray bars); $n = 16$–24; $^{**}p < 0.01$, $^{***}p < 0.001$ uninjured versus injured. **(B,C)** Neurospheres were harvested after 10 DIV, dissociated, and plated at clonal density. Secondary neurospheres were allowed to differentiate for 10–14 days. Immunostaining of secondary spheres confirmed that they contained NSCs that differentiated into several cell types including oligodendrocytes (Olig 2), astrocytes (GFAP), and neurons (β-tubulin). Cell nuclei were counterstained with DAPI. Scale bar = 50 μm. CCI, cortical contusion injury; GFAP, glial fibrillary acidic protein; NSC, neural stem cell.
